# Survey of enterovirus infections from hand, foot and mouth disease outbreak in china, 2009

**DOI:** 10.1186/1743-422X-8-508

**Published:** 2011-11-06

**Authors:** Fan Yang, Ting Zhang, Yongfeng Hu, Xiaofang Wang, Jiang Du, Yufen Li, Shaoxia Sun, Xiuhua Sun, Zhifang Li, Qi Jin

**Affiliations:** 1 State Key Laboratory for Molecular Virology and Genetic Engineering, Institute of Pathogen Biology, Chinese Academy of Medical Sciences, Beijing, People's Republic of China; 2 Institute for Viral Disease Control and Prevention, Chinese Center for Disease Control and Prevention, Beijing, PRC; 3 LinYi People's Hospital, Linyi, Shandong, PRC; 4 Heze Center for Disease Control and Prevention, Heze, Shandong, PRC

**Keywords:** Hand, Foot and Mouth Disease, enterovirus, human enterovirus 71, coxsackie virus A16; coxsackie virus B5

## Abstract

**Background:**

In China, a rapid expansion of Hand, foot, and mouth disease (HFMD) outbreaks has occurred since 2004 and HFMD has become an important issue for China. However, people are still only concerned with human enterovirus 71(HEV-71) and coxsackie virus A16 (CV-A16). Much of what is known about the other enterovirus infections relies on fractional evidence and old epidemic data, with little knowledge concerning their distribution. To alert potential threatens of the other enteroviruses, our study genetically characterized specimens from different regions of China and yielded novel information concerning the circulating and phylogenetic characteristics of enteroviral strains from HFMD cases.

**Findings:**

A total of 301 clinical throat swabs were randomly obtained from patients suffering from HFMD from the southern, northern and central regions of China during outbreaks in 2009. 266 of 301 (88.4%) HFMD cases were found positive for HEV and seven genotypes, HEV-71, CV-A16, -B5, -A4, -A6, -A10, and -A12, were detected.

**Conclusions:**

The HFMD pathogen compositions in the different regions of China were significantly different. HFMD epidemics might persist for a long time in China due to the multiple pathogen compositions, the enteroviral characteristic of recombination and co-infection, the ever-increasing travel and migration and the deficiency of effective vaccine. Our study deserves the attention on HFMD control and vaccine development.

## Background

Hand, foot, and mouth disease (HFMD) is a common childhood illness characterized by fever and vesicular eruptions on the hands, feet and mouth. HFMD is caused by a few serotypes of enteroviruses, most frequently coxsackie virus A16 (CV-A16) and human enterovirus 71 (HEV-71), and the severe forms were often associated with HEV71. Other enteroviruses (CV-A2, -A4, -A5, -A6, -A8, -A9,-A10, -A16,-B3 and -B5), may also be associated with HFMD outbreaks, sporadic cases or asymptomatic infections [1-3].

In recent years, the prevalence of HFMD in the Asia-Pacific region, especially in Southeast Asia, has greatly increased. In China, a rapid expansion of HFMD outbreaks has occurred since 2004 [4-7]. In 2009, the Chinese Center for Disease Control and Prevention (China CDC) confirmed 1,155,525 cases in Mainland China including 353 deaths. (http://www.moh.gov.cn/publicfiles/business/htmlfiles/mohbgt/s10639/201002/46043.htm; Chinese website).

### Objective

Although HFMD has become an important issue for China, people are still only concerned with HEV-71 and CV-A16. Much of what is known about the other enterovirus infections in China relies on fractional evidence and old epidemic data, with little knowledge concerning their distribution. To alert potential threatens of the other enteroviruses, our study genetically characterized specimens from different regions of China and yielded novel information concerning the circulating and phylogenetic characteristics of enteroviral strains from HFMD cases.

### Study design

A total of 301 clinical throat swabs were randomly obtained from patients suffering from HFMD in Guangdong (southern China), Beijing (northern China) and Shandong (central and eastern China) during outbreaks in 2009. All patients were identified according to the Ministry of Health diagnostic criteria (http://www.moh.gov.cn/publicfiles/business/htmlfiles/mohyzs/s3586/201004/46884.htm; Chinese website). The age distribution of HFMD cases was from 1 month to 12 years, and the median age was 2 years.

Semi-nested RT-PCR on the 5' partial region of VP1 with sense (GCIATGYTIGGIACICAYRT; *CCAGCACTGACAGCA*GYNGARAYNGG) and antisense (CICCIGGIGGIAYRWACAT; *TACTGGACCACCTGG*NGGNAYRWACAT) primers was used to type HEV directly from specimens as previously described [8]. Amplified DNA was purified using a commercial procedure (QIAquick Gel Extraction Kit (Cat. No 28704), Qiagen, Valencia, CA) and then sequenced on an ABI3730 automated sequencer (Applied Biosciences, Foster City, CA) using Big Dye reagents (version 3.0). Using the MEGA4 software, partial sequences were aligned with the sequences retrieved from GenBank by using the neighbor-joining method.

## Results

266 of 301 (88.4%) HFMD cases were found positive for HEV by using enterovirus general primers. HEV-71, CV-A16 occupied 50.4% and 38.3% of typed strains respectively. CV-B5 only detected from Shandong specimens and occupied 14.4 of Shandong typed strains. Additionally, four other genetically distinct strains, CV-A4, A6, A10, and A12, were also detected (Table 1). The 112 obtained sequences have been submitted to GenBank under accession numbers HM447216-447233 and HM459721-459814.

**Table 1:** Virological data from reported HFMD cases in China in 2009.

Province	HMFD cases	Positive for HEV	HEV-71	CV-A16	CV-A4	CV-A6	CV-A10	CV-A12	CV-B5
Beijing	85	75 (88.2%)	38 (50.7%)	31 (41.3%)	0 (0%)	0 (0%)	2 (2.7%)	4 (5.3%)	0 (0%)
Shandong	110	97 (88.2%)	60 (61.9%)	16 (16.5%)	0 (0%)	3 (3.1%)	1 (1.0%)	3 (3.1%)	14 (14.4%)
Guangdong	106	94 (88.7%)	36 (38.3%)	55 (58.5%)	3 (3.2%)	0 (0%)	0 (0%)	0 (0%)	0 (0%)
Total	301	266 (88.4%)	134 (50.4%)	102 (38.3%)	3 (1.1%)	3 (1.1%)	3 (1.1%)	7 (2.6%)	14 (5.3%)

**Figure 1:**
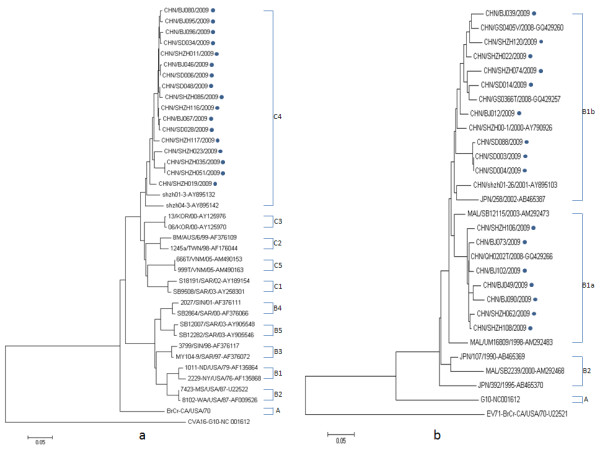
**Consensus phylogenetic tree constructed with human enterovirus (HEV)-71 (a) and coxsackie virus (CV)-A16 (b) sequences, including isolates from the present study (dots)**. The nucleotides correspond to the prototype BrCr strain at positions 2597-2900 for (a) and (b).

All the identified HEV-71 strains belonged to subgenogroup C4 (Figure 1a). High homologous (95.1%-100.0%) was noticed between the isolated stains from three different regions, but lower homologous showed between the new isolated stains and C4 previous strains isolated in 2001 and 2003. Notably, C4 is predominantly responsible for almost all HEV-71 infections in more than 10 years in Mainland China [9]. In contrast, the analysis of recent and previous HEV71 isolates in the Western Pacific Region showed that several subgenogroups, B1, B2, B3, B4, C1, C2, C3 and C4, were co-circulating in Australia, Malaysia, Singapore, Taiwan and Japan, respectively [3,10]. It seemed that the HEV71 strains evolved independently in Mainland China.

All CV-A16 strains evaluated in this study grouped with cluster B1a and B1b with 87.8-100% identity (Figure 1b), consistent with Chinese CV-A16 strains isolated between 1999 and 2008, which were also found in Taiwan, Malaysia, Thailand, Australia, Vietnam, and Saudi Arabia [11]. It indicated that the Chinese CV-A16 strains coevolved and co-circulated with those from surrounding countries. Interestingly, this was very different with Chinese HEV71 (the genetic evolution of Chinese HEV71 didn't share with neighboring countries), although both of them were genetically closely related and shared the similar transmission mode.

CV-B5 was present in 5.3% of typed strains with 98.4-100% identity. The Chinese strains [GenBank: AY695109], which were isolated from Zhejiang in 2002, were the closest observed among the strains analyzed. Strains clustering in the same subgroup were from Shandong of China, Korea, France, and Belarus, and all were isolated in 2005. Sequences from the 2000 Chinese Yunnan strain and the 1998 Shandong strain clustered in another subgroup, suggesting that CV-B5 types probably vary with time rather than geographical distribution.

All CV-A4, -A6, -A10, and -A12 strains evaluated segregated into individual genetic clusters distinct from previously reported sequences. The CV-A4 Chinese 2008 Shandong [GenBank: GQ253372] and 2004Yunnan [GenBank: AB268278] strains were the most closely related strains to those isolated in our study. The Japanese CV-A6 strains from 1999, 2000, 2003, and 2005 showed the closest relationship with our new CV-A6 strains. The CV-A10 Chinese 2008 and 2009 Shandong [GenBank: GQ214173 and GU947787] strains were more closely related to those in our study than the earlier Shandong strains and the other Japanese, German and American strains. We found only four sequences that could be compared with our three sequenced CV-A12 isolates, and the 2003 Japanese strains showed the closest relationship with our new CV-A12 strains.

## Discussion

The HFMD pathogen compositions in the southern, northern, and central regions of China were significantly different. Guangdong had an epidemic of HEV-71 over 10 years ago, but in 2009, CV-A16 was the dominant strain, likely as a result of the previous immune barrier on HEV-71. The epidemic of Beijing increased only in recent years, and both HEV-71 and CV-A16 were the major epidemic strains in 2009. Shandong reported the most HFMD cases and exhibited the most diverse pathogens, except HEV-71 and CV-A16, CV-B5 occupied 14.4% of typed strains in Shandong in 2009, this matched with its huge population and central communicating location.

In our study, we detected and analyzed four other HEVs (CV-A4, -A6, -A10, and -A12) that were simultaneously coexisting in China. Although some HFMD outbreaks and sporadic cases have been associated with them, eg., CV-A6 had been identified as the etiologic agent of HFMD outbreak in Finland [2], they only were detected from few HFMD patients in our study. Therefore, their relationship with HFMD may not be identified in China in 2009. However, the coexisting of multiple enteroviruses and the viral ability of co-infection and recombination suggested that, except HEV-71 and CV-A16, other enteroviruses also had the possibility to cause HFMD epidemic in China. It deserves our attention on HFMD control and vaccine development. HFMD epidemics might persist for a long time in China due to the multiple pathogen compositions, the enteroviral characteristic of recombination and co-infection, the ever-increasing travel and migration and the deficiency of effective vaccine.

## Abbreviations

HFMD: Hand, foot, and mouth disease; HEV: human enterovirus; CV: coxsackie virus.

## Competing interests

The authors declare that they have no competing interests.

## Authors' contributions

FY did laboratory testing, analyzed the test results, wrote and edited the manuscript. TZ, YH and XW did laboratory testing and co-wrote the manuscript. YL, SS, XS and ZL collected the samples and did laboratory testing. QJ is the leader of the study group and organized the overall project. All the authors read and approve the final manuscript.

## References

[B1] Blomqvist S Klemola P Kaijalainen S Paananen A Simonen ML Vuorinen T Roivainen M Co-circulation of coxsackieviruses A6 and A10 in hand, foot and mouth disease outbreak in Finland J Clin Virol 2010 48 1 49 54 10.1016/j.jcv.2010.02.002 20189452

[B2] Osterback R Vuorinen T Linna M Susi P Hyypiä T Waris M Coxsackievirus A6 and hand, foot, and mouth disease, Finland Emerg Infect Dis 2009 15 1485 1488 19788821 10.3201/eid1509.090438 PMC2819858

[B3] Chan KP Goh KT Chong CY Teo ES Lau G Ling AE Epidemic hand, foot and mouth disease caused by human enterovirus 71, Singapore Emerg Infect Dis 2003 9 1 78 85 12533285 10.3201/eid1301.020112 PMC2873753

[B4] Li L He YQ Yang H Zhu J Xu X Dong J Zhu Y Jin Q Genetic Characteristics of Human Enterovirus 71 and Coxsackievirus A16 Circulating from 1999 to 2004 in Shenzhen, People's Republic of China J Clin Microbiol 2005 43 3835 3839 10.1128/JCM.43.8.3835-3839.2005 16081920 PMC1233905

[B5] Yang F Jin Q He YQ Li L Hou Y The complete genome of Enterovirus 71 China strain Sci China C Life Sci 2001 44 178 183 10.1007/BF02879323 18726435

[B6] Yang F Ren LL Xiong ZH Zhao R He Y Bu G Zhou S Wang J Qi J Enterovirus 71 outbreak in the People's Republic of China in 2008 J Clin Microbiol 2009 47 2351 2352 10.1128/JCM.00563-09 19439545 PMC2708525

[B7] Zhang Y Tan XJ Wang H Yan DM Zhu SL Wang DY Ji F Wang XJ Gao YJ Chen L An HQ Li DX Wang SW Xu AQ Wang ZJ Xu WB An outbreak of hand, foot, and mouth disease associated with subgenotype C4 of human enterovirus 71 in Shandong, China J Clin Virol 2009 44 262 267 10.1016/j.jcv.2009.02.002 19269888

[B8] Nix WA Oberste MS Pallansch MA Sensitive, semi-nested PCR amplification of VP1 sequences for direct identification of all enterovirus serotypes from original clinical specimens J Clin Microbiol 2006 44 2698 2704 10.1128/JCM.00542-06 16891480 PMC1594621

[B9] Tan XJ Xu WB Molecular Epidemiological Research on Enterovirus 71 Chinese Journal of Vaccines and Immunization 2008 4 361 367

[B10] Wang SM Lei HY Su LY Wu JM Yu CK Wang JR Liu CC Cerebrospinal fluid cytokines in enterovirus 71 brain stem encephalitis and echovirus meningitis infections of varying severity Clin Microbiol Infect 2007 13 7 677 682 10.1111/j.1469-0691.2007.01729.x 17441979

[B11] Zhang Y Wang D Yan D Zhu S Liu J Wang H Zhao S Yu D Nan L An J Chen L An H Xu A Xu W Molecular evidence of persistent epidemic and evolution of subgenotype B1 coxsackievirus A16-associated hand, foot, and mouth disease in China J Clin Microbiol 2010 48 619 622 10.1128/JCM.02338-09 20018819 PMC2815627

